# Risk factors for development of personal protective equipment induced headache: e-survey of medical staff in Baltic states

**DOI:** 10.1186/s12913-022-08412-5

**Published:** 2022-08-10

**Authors:** Mantas Jokubaitis, Reda Timofejavaitė, Mark Braschinsky, Linda Zvaune, Alo-Rainer Leheste, Laura Gribuste, Paula Mattila, Sintija Strautmane, Austėja Dapkutė, Kristina Ryliškienė

**Affiliations:** 1 Centre of Neurology, Vilnius University, Vilnius, Lithuania; 2 Neurology Clinic, Tartu University Hospital, Tartu, Estonia; 3 Department of Neurology and Neurosurgery, University of Tartu, Tartu, Tartu, Estonia; 4 Department of Neurology and Neurosurgery, Riga East Clinical University Hospital, Riga, Latvia; 5 Department of Neurology, Pauls Stradins Clinical University Hospital, Riga, Latvia; 6 Faculty of Medicine, University of Tartu, Tartu, Estonia; 7 Faculty of Residency, Riga Stradins University, Riga, Latvia

**Keywords:** Personal protective equipment, Headache, Medical personnel

## Abstract

**Background:**

The COVID-19 pandemic led to an unprecedented increase in the use of personal protective equipment (PPE) among medical personnel. The goal of this study was to determine the risk factors and frequency of PPE-induced headache during the COVID-19 pandemic.

**Methods:**

From January 25 to March 1, 2021, an anonymous online survey was undertaken in the Baltic states.

**Results:**

In total, 2132 individuals participated. 52.3% experienced a PPE-induced headache. Usual onset time was between 2–3 h, lasting up to 1 h after PPE removal. The most common localization was in temporal and frontal regions. Headache usually occurred 2 to 3 days per week with an average pain score of 5.04 ± 1.80 points. Higher risk was associated with discomfort/pressure OR = 11.55, heat stress OR = 2.228, skin conditions OR = 1.784, long PPE use (duration 10-12 h) OR = 2,18, headache history prior PPE use OR = 1.207. Out of 52.3% respondents with PPE-induced headache, 45.5% developed de novo headache, whereas 54.5% had headache history. Statistically significant differences of PPE-induced headache between respective groups included severity (4.73 vs 5.29), duration (≥ 6 h 6.7% vs 8.2%), accompanying symptoms (nausea (19.3% vs 25.7%), photophobia (19.1% vs 25.7%), phonophobia (15.8% vs 23.5%), osmophobia (5.3% vs 12.0%)) and painkiller use (43.0% vs 61.7%).

**Conclusions:**

Over half of the medical personnel reported headache while using PPE. The risk was higher in individuals with headache history, increased duration of PPE use and discomfort while using PPE. Predisposed individuals reported PPE-induced headache which persisted longer, was more intense and debilitating than in the respondents with de novo headache.

**Supplementary Information:**

The online version contains supplementary material available at 10.1186/s12913-022-08412-5.

## Background

Personal protection equipment (PPE)-induced headache is a headache disorder that arises from prolonged compression of the pericranial soft tissues caused by protective equipment such as respirators, face masks, and eyewear [1]. PPE-induced headache is not a new type of headache disorder. It has already been described in the context of other professionals that wear equipment which creates pericranial tissue compression, such as goggles or helmets worn by swimmers, police officers, and others [2]. Due to a clear causative agent and simple treatment and prevention, this headache disorder was rarely mentioned in the medical literature and only isolated cases or studies with small sample sizes were described. During the COVID-19 pandemic, the use of PPE became widespread since it is an indispensable tool for preventing infection. Increased use of PPE during a COVID-19 pandemic allowed for a better understanding of PPE-induced headache and its characteristics. The identification of risk factors for PPE-induced headache could allow them to be addressed, thus reducing the impact of this headache disorder on medical personnel who rely on PPE use to safely care for COVID-19 patients. In this study we hypothesized that healthcare workers who experienced headache prior to the mandated use of PPE were more likely to develop PPE-induced headache.

## Methods

### Survey development, administration and ethics

During the survey development, we have reviewed the up-to-date literature on PPE-induced headache [1, 3]. We created a self-administered questionnaire comprised of 3 main sections in order to acquire the following: a) demographic information (age, sex, occupation and current department); b) headache history before mandatory PPE use (frequency, severity (measured using numeric pain rating scale), average duration, localization, accompanying symptoms, acute medication use, headache impact on working efficiency/sick leaves, diagnosis of primary headache disorder); c) headache associated with the PPE use (changes in headache characteristics after mandatory PPE use, types of PPE used, average use duration, other symptoms while using PPE, personal views on how likely PPE are causing/aggravating headache attacks, headache frequency, severity, localization, accompanying symptoms, time to onset of headache and when it stops, impact on working efficiency/sick leaves, acute medication use, other factors that may have influenced headache, other health problems induced by PPE (discomfort/pressure while using PPE, heat stress, skin conditions (acne, contact dermatitis, skin abrasions itching)). Lastly, based on presence or absence of headache history prior to the use of PPE, groups of “predisposed PPE-induced headache” and “de novo PPE-induced headache” were identified.

An advisory panel of neurologists from each Baltic State (Lithuania, Latvia and Estonia) assessed the initial draft of the survey to ensure its functionality and applicability. Some small improvements were made during the assessment. To confirm that the survey was integral, a group of healthcare personnel working in COVID-19 department in Vilnius University hospital Santaros klinikos completed a pilot version. Since it was regarded as thorough and simple to complete, no additional changes were made. The final anonymous online questionnaire was distributed through healthcare organizations like general practitioners’, nurses’ and medical students’ associations, medical students’ interest groups and via social network groups dedicated to healthcare workers. Participants were included in the study if they worked in a healthcare institution and wore head and/or face PPE. The study was conducted in the Baltic States from January 25 to March 1, 2021.

The survey completion was voluntary, and no financial incentive was received by the study participants. None of the involved healthcare organizations received any funding for the distribution of the survey. Since the data acquired was anonymous and without the ability to identify a specific person, no ethics approval was sought. Ethics approval was deemed unnecessary by Vilnius Regional Biomedical Research Ethics Committee with respect to the General Data Protection Regulation Principle 26. All participants consented to participate by marking confirmation in the e-survey that they agree to the use of their anonymous data for scientific publication. According to Article 2 of the Republic of Lithuania's Law on Ethics of Biomedical Research, no particular informed consent was necessary, as affirmed by the Vilnius Regional Biomedical Research Ethics Committee, since anonymous surveys are not considered biomedical research.

### Statistical analysis

Descriptive analyses were used to study baseline characteristics. Variables that were measured on the ordinal scale were compared using a Mann–Whitney U test and summarized using median. Interval level data were compared using a t test and described using mean (standard deviation). Chi-square analyses were used to compare nominal demographic data and PPE-associated headache characteristics across 2 groups (respondents with de novo and predisposed PPE-associated headache). Binomial logistic regression analyses were performed to identify the independent variables associated with the development of PPE-associated headache. Additional corrections for age and gender were made. The modification was undertaken to ensure that the results were not influenced by sample inequities, such as a large proportion of women and widely varying age of the respondents. Predictor variables that were significant at *P* < 0.05 were retained in the model. Statistical significance was set at *P* < 0.05. All statistical analyses were performed using the SPSS statistical package program version 26.0 for Windows and MS Excel 2019.

## Results

In total, 2132 individuals participated in the study, of those 88.37% were female. The age of respondents varied between 18 and 70 years with the average of 40.3 and median of 38 years. 24.28% (221) were working COVID-19 department at the time of survey completion. Table 1 depicts demographic data of study participants.

**Table 1:** Demographic data and headache history among participants in this study

	Overall	Lithuania	Latvia	Estonia	*p* value
	n (%)	n (%)	n (%)	n (%)	
Respondents	2132 (100.00)	910 (42.68)	488 (22.89)	734 (34.43)	-
Age	40.33 (13.07)^a^	37.42 (11.59)^a^	44.14 (14.33)^a^	41.41 (13.14)^a^	< 0.001
Female	1884 (88.37)	800 (87.91)	430 (88.11)	654 (89.10)	-
Occupation:					< 0.001
Nurse	738 (34.62)	336 (36.92)	75 (15.37)	327 (44.55)	-
Medical doctor	731 (34.29)	249 (27.36)	279 (57.17)	203 (27.66)	-
Resident	255 (11.96)	104 (11.43)	65 (13.32)	86 (11.72)	-
Nurse assistant	104 (4.88)	46 (5.05)	7 (1.43)	51 (6.95)	-
Medical student	67 (3.14)	25 (2.75)	8 (1.63)	34 (4.63)	-
Dentists/dentists assistants	51 (2.39)	49 (5.38)	1 (0.20)	1 (0.14)	-
Volunteer	47 (2.20)	44 (4.84)	-	3 (0.41)	-
Paramedic	26 (1.22)	7 (0.77)	10 (2.05)	9 (1.23)	-
Other^b^	113 (5.30)	40 (5.49)	43 (4.73)	20 (2.72)	-
Headache before PPE^c^ use:	1085 (50.89)	365 (40.11)	271 (55.53)	449 (61.17)	< 0.001
Headache disorder diagnosis^d^	339 (15.90)	91 (10.00)	87 (17.83)	161 (21.93)	0.003
Tension-type headache	174 (8.16)	30 (3.30)	57 (11.68)	87 (11.85)	< 0.001
Migraine without aura	105 (4.92)	38 (4.17)	15 (3.07)	52 (7.08)	0.025
Migraine with aura	98 (4.59)	22 (2.42)	23 (4.71)	53 (7.22)	0.016
Cluster headache	5 (0.23)	-	1 (0.20)	4 (0.54)	0.178
Secondary headache disorders	20 (0.93)	9 (0.99)	6 (1.23)	5 (0.68)	0.191

^a^ Mean (standard deviation)

^b^ Other—physiotherapist, occupational therapist, laboratory worker, medical psychologist, medical biologist, radiology technologist etc.

^c^ *PPE* Personal protective equipment

^d^ Number of participants with at least one headache diagnosis (some participants experienced more than one type of headache)

**Fig. 1:**
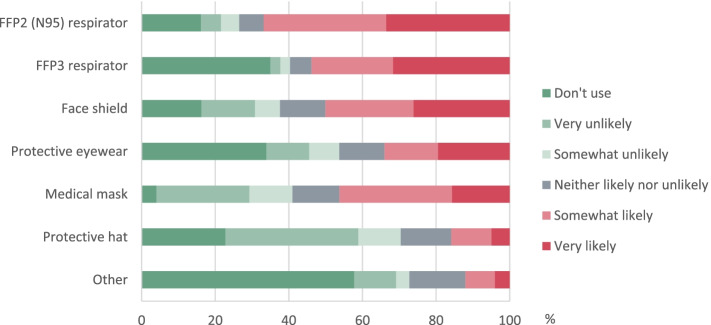
Respondents’ answers to the question “Which head and face personal protective equipment do you think causes/provokes headaches?”

52.3% (*n* = 1115) of the respondents experienced a PPE-induced headache. An average numeric pain rating scale score was 5.04 ± 1.80 points. The need for rest was indicated in 65.3% (*n* = 728), while the pain was rarely accompanied by photophobia (22.7%, *n* = 253), phonophobia (20.0%, *n* = 223), nausea (22.8%, *n* = 254), visual impairment (19.6%, *n* = 219), vomiting (1.7%, *n* = 19). The time to headache onset was 2 to 3 h in 43.4% of individuals, usually lasting up to 1 h after the removal of PPE (42.2%). The frequency of the headache occurrence was 2 to 3 days per week (34.0%). The most frequent localization of the pain due to PPE was in temporal (69.3%) and frontal (56.1%) regions of the head. Acute treatment was used by 53.1% of respondents, with non-steroidal anti-inflammatory drugs (including paracetamol) being the most common (93,4%). In contrast, combinations of analgesics with caffeine or codeine (19.3%) and triptans (3.9%) were used less often. 73.6% of the respondents have felt that their working efficiency was negatively affected, but only 2.2% have taken sick leave because of PPE induced headache. The most common responses given by medical personnel to the question "Which head and face PPE do you think causes/provokes headaches?" were FFP2, FFP3 respirators and face shield (Fig. 1).

Out of 52.3% of respondents who reported PPE-induced headache, 45.5% of individuals developed de novo headache, whereas 54.5% had previously experienced other types of headaches, and 32.4% of them had headache diagnosis (Table 1). Patients with a headache history indicated changes of previous headache disorder: increased frequency (61.0%), severity (48.5%), duration (46.1%), in addition to more frequent use of painkillers (46.7%). In addition, individuals with a previous headache history reported PPE-induced headache that lasted longer, was more severe, and was more frequently accompanied by additional symptoms as well as increased analgesic use (Table 2). Table 3 lists risk factors associated with PPE-induced headache (corrected for age and gender). Although analysis of Lithuanian data revealed that FFP2 respirators were a risk factor for headache, the finding became insignificant when all Baltic countries were examined together.

**Table 2:** The differences of headache characteristics between predisposed individuals with previous headache history and respondents who developed de novo headache while using personal protective equipment (PPE)

Headache characteristics	De novo headache (*n* = 507)		Predisposed headache (*n* = 608)		*p* value*
	n	%	n	%	
Start time:					< 0.001
within 3 h	342	67.45	345	56.74	
In 4–5 h	115	22.68	153	25.16	
in ≥ 6 h	50	9.86	110	18.09	
Headache stops after taking off PPE					0.001
Immediately	79	15.58	54	8.88	
in 1 h	204	40.24	219	36.02	
in 2–3 h	146	28.8	206	33.88	
In 4–5 h	44	8.68	79	12.99	
In ≥ 6 h	34	6.71	50	8.22	
Frequency					< 0.001
Almost every day	66	13.02	48	7.89	
4–5 days per week	49	9.66	40	6.58	
2–3 days per week	201	39.64	178	29.28	
1 day per week	102	20.12	170	27.96	
Less than 1 day per week	89	17.56	172	28.29	
Severity	4.73 (1.75)^a^		5.29 (1.79)^a^		< 0.001
Localization					
Orbital region	145	28.60	234	38.49	0.001
Occipital region	165	32.54	234	38.49	0.039
Temporal region	354	69.82	419	68.91	0.743
Frontal region	274	54.04	351	57.73	0.217
Nose	204	40.24	174	28.62	< 0.001
Cheeks	169	33.33	147	24.18	0.001
Accompanying symptoms					
Nausea	98	19.33	156	25.66	0.012
Vomiting	5	0.99	14	2.30	0.091
Photophobia	97	19.13	156	25.66	0.010
Phonophobia	80	15.78	143	23.52	0.001
Osmophobia	27	5.33	73	12.01	< 0.001
Visual impairment	113	22.29	105	17.27	0.035
Need for rest	324	63.91	404	66.45	0.375
Use of acute pain medication	218	43.00	375	61.68	< 0.001

^a^ Mean (standard deviation)

**Table 3:** Risk factors associated with personal protective equipment (PPE)-induced headache corrected for age and gender

Risk factor	Odds ratio	*p* value	95% C.I
Discomfort/pressure while using PPE	12.11	< 0.001	8.88; 16.53
Heat stress	2.23	< 0.001	1.80; 2.76
Skin conditions	1.65	< 0.001	1.34; 2.05
Average PPE use duration: (reference 1–3 h)		0.003	
4–6 h	1.49	0.001	1.18; 1.87
7–9 h	1.13	0.516	0.79; 1.62
10–12 h	2.18	0.012	1.19; 3.99
> 12 h	1.49	0.217	0.79; 2.80
Headache before PPE use	1.21	0.073	0.98; 1.48
Female gender	1.79	< 0.001	1.29; 2.49
Age (reference 18–29 years old)		< 0.001	
30–44 years old	0.68	0.004	0.52; 0.89
45–59 years old	0.47	< 0.001	0.36; 0.61
≥ 60 years old	0.25	< 0.001	0.17; 0.38

## Discussion

The present study demonstrates the burden of PPE-induced headaches among medical personnel during the COVID-19 pandemic in the Baltic states. The strongest risk factors associated with PPE-induced headaches were increased pressure on the head, skin lesions, thermal stress, prolonged use and previous headache history. The recent meta-analysis and systematic review of adverse outcomes associated with PPE use among health workers by Galanis et al. yielded similar results [4]. The most common adverse event reported was headache. The study discovered that the longer the duration of PPE wearing, the greater the probability of adverse events. In addition, wearing PPE on consecutive days was also a risk factor. Our finding that younger age and female gender was associated with an increased risk of headache is consistent with the results of the majority of the studies included in the meta-analysis [4].

The hypothesis of our study was confirmed as we found that more than half of the medical workers experienced a PPE-induced headache, with the risk being higher among those who had a prior headache history. Individuals with a previous headache history (most commonly tension-type headache) reported PPE-induced headache that lasted longer, was more severe, and more commonly associated with accompanying (nausea, photophobia, phonophobia, osmophobia) and additional symptoms (visual impairment) as well as higher analgesic use. In the study by Yuksel et al. it was found that PPE (particularly the use of masks and respirators) was associated with significant worsening of migraine in both non-healthcare and healthcare workers, although being a health worker was an independent risk factor for migraine worsening [4, 5].

The PPE-induced headache does not completely fit the criteria of external-compression headache as defined by the third edition of the International Classification of Headache Disorders (ICHD-3) [6]. In addition to pure mechanical pressure and direct activation of skin nociceptors other complex factors such as hypercapnia and the ensuing cerebral vasodilatation caused specifically by masks and respirators might contribute to the development of this headache disorder. Conversely, while some data support hypercapnia as a possible pathophysiologic mechanism of a PPE-induced headache, given the lack of a performance metric and the profound intricacies underlying headache pathophysiology in general, the findings of these studies should be interpreted with caution [7, 8]. However, while the mechanisms underlying PPE-induced headache are not yet fully understood, the clinical data presented in this study, as well as data that has accumulated in the literature during COVID-19 pandemic, provide a substantial basis for considering PPE-induced headache to be classified as a secondary headache disorder in future editions of the ICHD.

It is unsurprising that a PPE which causes discomfort and headaches is worn incorrectly, therefore reducing its effectiveness. According to the study of Rebmann et al., it was found that PPE discomfort was the second most common cause for taking off the PPE [9]. Furthermore, violations of safe PPE use i.e., touching the respirator, adjusting its position, etc., occurred 25.7 times on average during a 12-h shift [9]. As a result, it is advised to wear PPE that is the correct size and to take breaks to remove PPE during work.

It should be noted that, according to our research, only 15.9% of medical workers have been diagnosed with a headache disorder, while 50.9% have had a previous headache history. It is a significant problem since an undiagnosed headache can result in self-medication and inadequate pain control. This is especially true for healthcare workers, who are more likely to suffer from a primary headache disorder as a result of their heavy workloads, frequent stressful situations, and night shift work [10].

Between the Baltic countries, there were some statistically significant differences. Lithuanian healthcare workers reported more frequent and severe headaches, as well as greater skin injury from PPE. Additionally, participants from Lithuania who completed the survey were shown to have a higher rate of worsening of preexisting headache disorder and, as a result, a higher use of analgesics. PPE use, on the other hand, had a greater influence on working efficiency and sleep disturbances among employees in Estonia, while personnel in Latvia took sick leave more frequently than workers from the rest of the countries.

The strengths of our study are large sample size and inclusion of various groups of hospital staff. Nevertheless, there are few limitations associated with our study. Firstly, the survey was conducted online and is prone to non-response bias. Additionally, due to the study's self-administered and retrospective design, there is a chance of recall bias. Also, individuals suffering from headaches may have been more willing to complete the survey than those who did not. Because the respondents were primarily young individuals (mean age of 40 years), older respondents and those with limited knowledge of information technology may have been excluded. Furthermore, the majority of the respondents were female. It is well known that women are more likely to experience a primary headache, therefore, the uneven proportion of female participants could have slightly decreased the statistical power [11]. It should be noted that a recent COVID-19 infection in medical personnel during the study period could have had a small effect on study results. Finally we did not investigate some additional risk factors, such as night shift work, working hours, body mass index, depression, and anxiety, which could have influenced our study results.

## Conclusions

The results of this study confirm our hypothesis that individuals with a history of headaches prior to the mandatory use of PPE due to COVID-19 pandemic are at increased risk of developing a PPE-induced headache. Predisposed individuals reported PPE-induced headache that persisted longer, was more intense and debilitating than in the respondents who developed headache de novo. In addition, the higher rate of PPE-induced headache was observed in personnel who specified risk factors such as the length of PPE use, increased pressure on the head, skin lesions and thermal stress.

## Supplementary Information

**Additional file 1.**

## Data Availability

The raw data that support the findings of this study are available in Supplement 1.
